# Indian expert consensus on prophylactic incisional single-use negative pressure wound therapy in hip and knee arthroplasty: A modified Delphi study

**DOI:** 10.1051/sicotj/2026060

**Published:** 2026-09-22

**Authors:** Vaibhav Bagaria, Sachin Tapasvi, Anjali Tiwari

**Affiliations:** 1 Department of Orthopaedic Surgery, Sir H N Reliance Foundation Hospital Mumbai Maharashtra India; 2 The Orthopaedic Speciality Clinic Pune Maharashtra India; 3 Global Research Cell, Dr D.Y. Patil Dental College & Hospital, Dr D. Y. Patil Vidyapeeth (Deemed to Be University) Pune Maharashtra India

**Keywords:** Single-use negative pressure wound therapy, Incisional sNPWT, Total hip arthroplasty, Total knee arthroplasty, Surgical site infection

## Abstract

*Background:* Surgical site complications remain an important cause of morbidity following total hip and knee arthroplasty. While international guidelines support the selective use of prophylactic single-use negative pressure wound therapy (sNPWT) in appropriately selected high-risk patients and surgical incisions, these recommendations are largely derived from studies performed in highly standardized healthcare environments. Differences in patient characteristics, healthcare delivery, perioperative pathways, resource utilization, and postoperative surveillance may influence their applicability across diverse healthcare systems. Consequently, pragmatic, context-specific recommendations are needed to translate the available evidence into routine clinical practice in India and other countries with similar healthcare settings. *Methods***:** A modified Delphi process was conducted involving 10 experienced arthroplasty surgeons from across India. An exploratory meeting identified key clinical questions and developed candidate statements. Subsequently, nine experts independently evaluated 13 statements using anonymous four-point Likert-scale voting. Consensus was predefined as ≥75% agreement. *Results:* Twelve of the 13 candidate statements achieved consensus (agreement 77.8%–100%). The expert panel agreed that prophylactic sNPWT may be considered in selected high-risk situations, including revision arthroplasty involving implant removal, prolonged operative time (>120 min), morbid obesity (BMI > 40 kg/m^2^), uncontrolled diabetes (HbA1c > 8%), and high-tension wounds. Consensus was also achieved on key contraindications and practical application principles. These recommendations represent consensus-informed clinical guidance rather than definitive evidence-based recommendations. *Conclusion:* This modified Delphi consensus translates the available evidence and collective expert experience into practical recommendations for the selective use of prophylactic sNPWT following hip and knee arthroplasty. By providing a structured framework for patient selection and application, it aims to support consistent, evidence-informed decision-making in routine clinical practice, particularly in healthcare settings where context-specific implementation of international recommendations is required. Prospective clinical and health economic studies are needed to further validate these recommendations.

## 1 Introduction

Total hip and knee arthroplasty are highly successful procedures for alleviating pain and restoring function, leading to a substantial increase in their utilization across India. Data from the global and also Indian Society of Hip and Knee Surgeons (ISHKS) registry highlight this trend, with a dramatic rise in procedures recorded between 2006 and 2016 [1]. As the volume of arthroplasties grows, optimizing patient outcomes and minimizing complications, particularly surgical site infections (SSIs) and wound healing issues, becomes paramount. In this context, single-use negative pressure wound therapy (sNPWT), applied prophylactically to closed surgical incisions, has emerged as a promising strategy for reducing postoperative wound complications.

sNPWT functions by applying controlled sub-atmospheric pressure across a closed surgical incision through a sealed dressing connected to a portable suction device. This negative pressure stabilizes the wound edges, reduces lateral tension across the incision, and facilitates removal of exudate and hematoma formation. In addition, the therapy helps maintain a protected wound environment and may improve local perfusion and lymphatic drainage. These combined effects are thought to reduce wound complications such as persistent drainage, dehiscence, and SSI following major orthopedic procedures [2]. This approach is supported by international guidance from organisations such as the National Institute for Health and Care Excellence (NICE) and the World Health Organization (WHO), which suggest considering prophylactic sNPWT for appropriately selected patients and closed surgical incisions at increased risk of postoperative wound complications rather than recommending its routine use for all arthroplasty procedures [3, 4]. Further consensus from the World Union of Wound Healing Societies (WUWHS) also advocates for its use on closed incisions [5].

However, the direct application of these international guidelines to the Indian healthcare landscape is challenging. Significant differences exist in patient demographics, comorbidities, standards of care, healthcare infrastructure, and follow-up systems. There is a clear need for tailored guidance that addresses the specific clinical scenarios and resource considerations relevant to orthopaedic surgeons in India. SSI remains a key contributor to morbidity following arthroplasty. Although global benchmarks report SSI rates of approximately 1%–2% after total hip and knee arthroplasty, emerging Indian data indicate higher and more variable rates, with arthroplasty-specific SSI incidences reported between 2%–4% and overall orthopaedic SSI rates exceeding 5% in multicentric surveillance studies [6].

Therefore, this modified Delphi consensus was undertaken to translate the available evidence and collective clinical experience into pragmatic recommendations for the selective use of prophylactic sNPWT following total hip and knee arthroplasty in the Indian clinical setting. The objective was to establish consensus on patient selection, contraindications, and application principles that could support consistent, evidence-informed decision-making in routine arthroplasty practice, while providing a practical framework that may also be relevant to healthcare systems facing similar clinical and resource considerations.

## 2 Methodology

### 2.1 Study design

**Figure 1:**
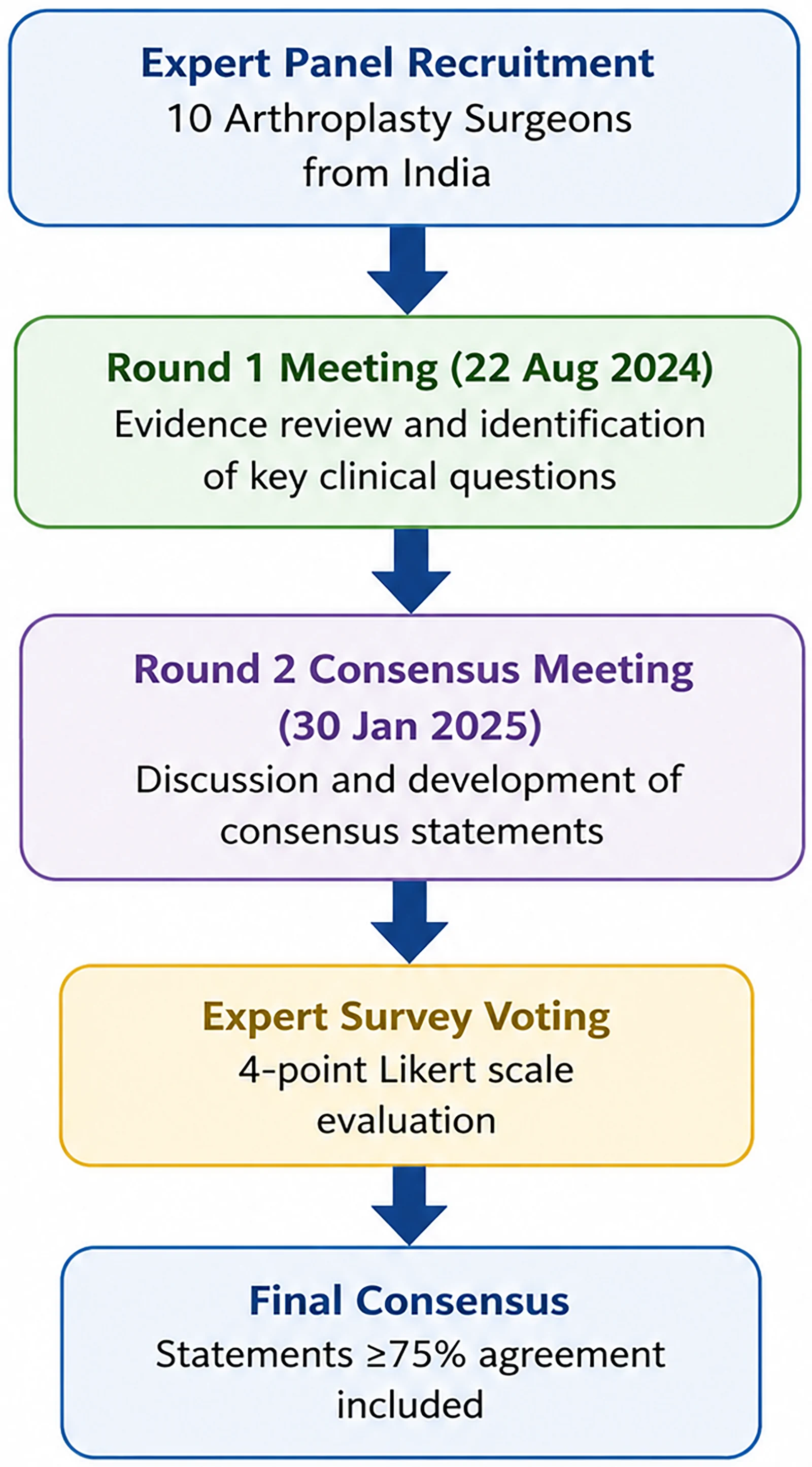
Flow diagram illustrating the modified Delphi consensus process used to develop recommendations on prophylactic single-use negative pressure wound therapy (sNPWT) for hip and knee arthroplasty.

This study employed a modified Delphi consensus methodology to develop practical recommendations for the use of prophylactic sNPWT following hip and knee arthroplasty. Unlike a classical Delphi process, which typically consists of multiple sequential rounds of anonymous questionnaires with controlled feedback, the modified Delphi approach combines structured expert discussion with anonymous voting. It is widely used in healthcare research when preliminary consensus building and contextual adaptation are required. The present methodology consisted of two sequential phases: an exploratory meeting to identify clinically relevant domains and formulate candidate statements, followed by a structured consensus meeting incorporating anonymous voting using predefined agreement criteria (Figure 1).

### 2.2 Development of consensus statements

The candidate consensus statements were developed through a structured process combining available scientific evidence with expert clinical experience. Prior to the exploratory meeting, a targeted literature review was conducted using PubMed, Google Scholar, and guideline documents from international organisations including the WHO, NICE, and the WUWHS. The retrieved evidence was used to identify clinically relevant themes and areas where uncertainty existed regarding prophylactic sNPWT in hip and knee arthroplasty. During the exploratory meeting, experts reviewed the available evidence, discussed variations in clinical practice within the Indian healthcare setting, and generated preliminary consensus statements. These statements were subsequently refined through group discussion before being presented for anonymous voting during the consensus meeting.

### 2.3 Expert panel

Ten orthopaedic surgeons with extensive experience in hip and knee arthroplasty were invited to participate in the consensus process. Experts were selected using predefined eligibility criteria to ensure clinical and academic expertise. Inclusion criteria comprised: (1) a minimum surgical volume of at least 100 primary or revision hip and knee arthroplasty procedures per year; (2) a minimum of 10 years of independent surgical experience in arthroplasty practice; and (3) demonstrated academic contribution to the field, defined as authorship of at least five peer-reviewed publications in orthopaedics, arthroplasty, or wound management. In addition, efforts were made to ensure representation across diverse geographic regions of India and practice settings. All participating surgeons had substantial experience managing complex primary and revision arthroplasty cases and were actively engaged in clinical decision-making related to perioperative wound management. None of the experts were involved in the manufacture, marketing, or commercial promotion of any sNPWT device during the consensus process. Selection of panel members was based solely on predefined clinical and academic eligibility criteria to minimise selection bias (Table 1).

**Table 1:** Characteristics of the expert panel participating in the orthopaedic council.

Expert	Region	Practice setting	Primary specialty	Years of experience*	Approximate annual arthroplasty volume*	Conflict of interest declaration*
Dr. Sudheer Karlakki	United Kingdom	Robert Jones and Agnes Hunt Orthopaedic Hospital	Hip & Knee Arthroplasty	>20 years	>300	None
Dr. Vaibhav Bagaria	Mumbai, India	Sir H. N. Reliance Foundation Hospital	Adult Reconstruction, Hip & Knee Arthroplasty	>20 years	>500	None
Dr. Sachin Tapasvi	Pune, India	Private Orthopaedic Practice	Knee Arthroplasty, Sports Medicine, Robotics, Revision Surgery	>25 years	>600	None
Dr. Hrushikesh Saraf	Pune, India	Private Orthopaedic Practice	Arthroscopy, Arthroplasty, Trauma	>20 years	>500	None
Dr. N Rajkumar	Coimbatore, India	Private Orthopaedic Practice	Hip & Knee Arthroplasty	>25 years	>500	None
Dr. Krishna Kiran	Hyderabad, India	Private Orthopaedic Practice	Orthopaedic Surgery	>20 years	>500	None
Dr. Rakesh Rajput	Kolkata, India	CMRI Hospital	Arthroplasty	>25 years	>500	None
Dr. Vijay Bose	Chennai, India	High-volume Arthroplasty Unit	Hip & Knee Arthroplasty	>23 years	>700	None
Dr. Subhash Jangir	Gurugram, India	Private Orthopaedic Practice	Arthroplasty	>20 years	>300	None
Dr. Shubhang Aggarwal	Jalandhar, India	NHS Hospital	Orthopaedic Surgery	>20 years	>300	None
Dr. Mukesh Laddha	Nagpur, India	Orthopaedic Practice	Orthopaedic Surgery	>20 years	>400	None

### 2.4 Expert identification and selection

Experts were identified and invited by the study investigators based on predefined eligibility criteria. Selection was independent of the study sponsor and aimed to achieve representation from different geographical regions of India, diverse hospital settings (academic institutions, private hospitals, and tertiary referral centres), and surgeons with substantial experience in both primary and revision hip and knee arthroplasty. The sponsor had no role in identifying, nominating, inviting, or selecting the expert panel.

Prior to participation, all experts completed conflict-of-interest declarations. None of the participating surgeons were employees of the sponsoring organisation or involved in the manufacture or commercial promotion of sNPWT devices. Any relevant professional relationships were disclosed and considered during the consensus process. Although the consensus meeting received industry funding for logistical support, all methodological decisions, expert selection, statement development, anonymous voting, and interpretation of findings were conducted independently of the sponsor to minimise potential bias.

#### 2.4.1 Phase 1: Exploratory meeting

An initial exploratory meeting was conducted on 22 August 2024 to identify key clinical challenges and practice variations related to the use of sNPWT in arthroplasty. All 10 invited experts participated in this phase.

During this session, open discussions were held to review existing literature and international guidance, identify gaps in evidence relevant to the Indian clinical context, and define key domains for consensus development. Based on these discussions, 13 candidate statements were formulated addressing three broad domains: Indications for sNPWT, Contraindications for sNPWT, technical considerations, and application principles.

#### 2.4.2 Phase 2: Consensus meeting and anonymous voting

A subsequent in-person consensus meeting was conducted on 30 January 2025. Nine of the 10 experts participated in this phase; one expert was unable to attend due to logistical and travel constraints. To minimise bias and ensure unbiased expert input, voting was conducted anonymously. All nine experts attending the consensus meeting completed voting for every candidate statement. Consequently, agreement percentages were calculated using a consistent denominator of nine participants for all consensus statements. The candidate statements developed during Phase 1 were presented and discussed in a structured round-table format. Following the discussions, participants independently rated their level of agreement with each statement using a 4-point Likert scale:

- Strongly agree
- Agree
- Disagree
- Strongly disagree.

A predefined consensus threshold of ≥75% agreement (combined “strongly agree” and “agree” responses) was used to determine acceptance of each statement. Voting was conducted anonymously using individual response forms to minimise dominance bias and encourage independent judgement. Only aggregated voting results were analysed. For complete transparency, the initial list of all 13 candidate statements, together with the number of responses for Strongly agree, Agree, Disagree, and Strongly disagree, the overall percentage agreement, and the final consensus outcome, are presented in Table Table 2.

**Table 2:** Complete Delphi voting results for all candidate consensus statements.

Statement No.	Candidate statement	Strongly agree (n)	Agree (n)	Disagree (n)	Strongly disagree (n)	Agreement (%)	Consensus outcome
S1	Revision arthroplasty involving implant removal is an indication for prophylactic sNPWT.	9	0	0	0	100	Accepted
S2	Arthroplasty procedures lasting >120 min should receive prophylactic sNPWT.	7	1	1	0	88.9	Accepted
S3	Patients with BMI >40 kg/m^2^ should receive prophylactic sNPWT.	6	2	1	0	88.9	Accepted
S4	Patients with HbA1c >8% should receive prophylactic sNPWT.	5	3	1	0	88.9	Accepted
S5	Routine use of sNPWT is recommended following bariatric surgery.	4	3	2	0	77.8	Accepted
S6	High-tension wounds requiring difficult primary closure should receive prophylactic sNPWT.	8	1	0	0	100	Accepted*
S7	sNPWT is contraindicated in wounds requiring dressing changes every 24 h because of heavy exudate.	6	2	1	0	88.9	Accepted
S8	Adhesive allergy is a contraindication to sNPWT.	5	2	2	0	77.8	Accepted
S9	The dressing should be applied with the knee flexed to approximately 90°.	7	1	1	0	88.9	Accepted
S10	The soft port should be positioned superiorly in an anti-gravity orientation.	9	0	0	0	100	Accepted
S11	Silicone-based adhesives are preferred over acrylic-based dressings.	8	1	0	0	100	Accepted*
S12	Routine second dressing changes are unnecessary unless clinically indicated.	9	0	0	0	100	Accepted
S13	Routine prophylactic sNPWT should be used in all primary hip and knee arthroplasty procedures regardless of patient risk profile.	3	3	2	1	66.7	Rejected

Consensus was predefined as ≥75% agreement (combined Strongly agree and Agree responses). Nine experts participated in the anonymous voting. One statement (S13) failed to achieve the predefined consensus threshold and was therefore excluded from the final recommendations.

### 2.5 Consensus definition

Statements achieving ≥75% agreement were considered to have reached consensus and were included as final recommendations. Statements failing to reach this threshold were excluded from the final consensus set.

### 2.6 Literature support

**Figure 2:**
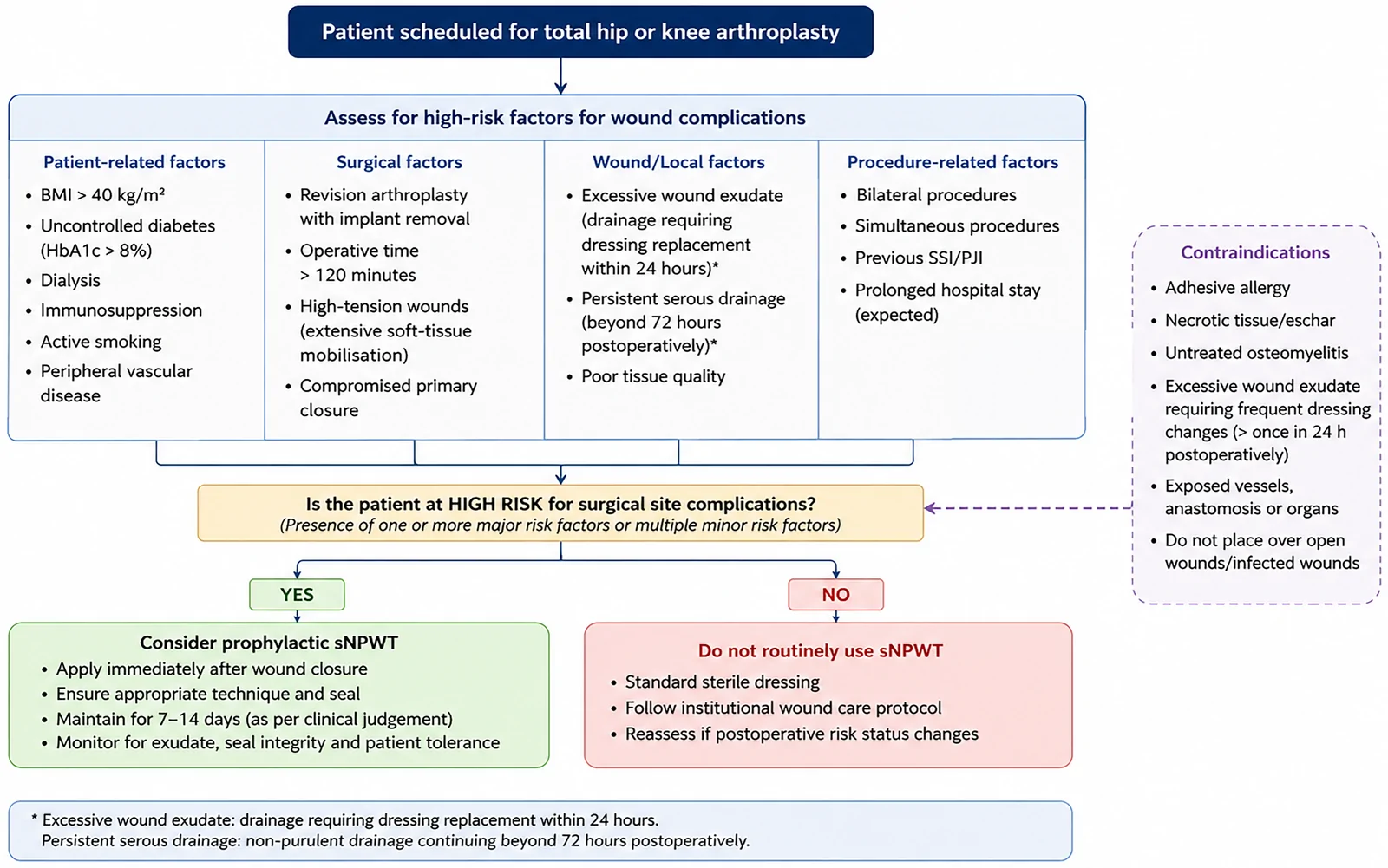
Clinical decision algorithm for prophylactic sNPWT use in arthroplasty

A targeted literature search was conducted to support the development and interpretation of consensus statements (Figure 2). Electronic databases including PubMed and Google Scholar were searched using combinations of the following keywords:

- Negative pressure wound therapy
- Incisional SNPWT
- Single-use SNPWT
- Total hip arthroplasty
- Total knee arthroplasty
- Surgical site infection
- Wound complications.

Relevant studies, systematic reviews, and international guideline documents were reviewed and used to contextualize the consensus recommendations.

### 2.7 Evidence supporting consensus statements

The literature identified during the targeted review was used to support discussion of each candidate statement and provide clinical context during the consensus process. However, formal evidence grading using systems such as GRADE was not performed because the objective of the study was to develop expert consensus recommendations rather than evidence-based clinical practice guidelines. Accordingly, the final recommendations should be interpreted as consensus-informed guidance supported by the available literature rather than high-level evidence-based recommendations.

### 2.8 Evidence rating framework

To improve the interpretation of the consensus recommendations, each accepted statement was assigned a simplified evidence level based on the highest quality of supporting published literature available during the consensus process. Because this study was designed as a modified Delphi consensus rather than a formal evidence-based clinical practice guideline, a full GRADE assessment was not performed. Instead, evidence levels were adapted from the Oxford Centre for Evidence-Based Medicine and previous orthopaedic consensus publications. *Level A* represents high-quality evidence supported by systematic reviews, meta-analyses, randomised controlled trials, or international clinical guidelines; *Level B* represents moderate-quality evidence derived from prospective or retrospective comparative studies and consistent observational evidence; and *Level C* represents limited evidence supported primarily by expert consensus, indirect evidence, biological rationale, or clinical experience where arthroplasty-specific evidence remains limited.

### 2.9 Data analysis

Responses were analysed descriptively. Agreement for each statement was calculated as the percentage of experts selecting either “Agree” or “Strongly agree”. No inferential statistical analyses were performed because the objective of the study was to determine expert consensus rather than evaluate treatment effectiveness. Statements achieving ≥75% agreement were accepted as consensus recommendations. Agreement for each statement was calculated as the percentage of participants selecting either “Strongly agree” or “Agree.” Consensus agreement across the finalized statements ranged from 77.8% to 100%.

### 2.10 Operational definitions of clinical terms

To improve consistency in the interpretation of the consensus statements, the expert panel agreed on practical operational definitions for commonly used clinical terms.

*High-tension wound:* A surgical incision in which primary closure is achieved only after considerable soft-tissue tension, requiring forceful approximation of wound edges, extensive soft-tissue mobilisation, or where blanching of the wound margins is observed after closure. Examples include revision arthroplasty with extensive soft-tissue dissection, obese patients with thick subcutaneous tissue, and wounds demonstrating excessive tension during flexion testing.

*Compromised (less-than-appropriate) primary closure:* Primary wound closure in which satisfactory edge approximation cannot be achieved without excessive tension, multiple reinforcing sutures, residual wound gapping, or concern regarding tissue viability or perfusion as determined by the operating surgeon.

*Excessive wound exudate:* Drainage sufficient to saturate the dressing within 24 h or requiring dressing replacement more frequently than every 24 h despite appropriate wound closure and the absence of major postoperative bleeding.

*Persistent serous drainage:* Continued non-purulent wound drainage beyond 72 h after surgery or drainage requiring repeated dressing changes after the expected immediate postoperative period. Persistent drainage should prompt careful clinical assessment to exclude early SSI or wound dehiscence before considering prophylactic sNPWT.

These operational definitions are summarised in the footnotes to Table 3 and were applied consistently throughout the consensus process.

**Table 3:** Expert consensus statements and agreement rates on the preemptive use of sNPWT in hip and knee arthroplasty.

Domain	Consensus statement no.	Consensus statement	Evidence level*	Agreement rate (%)
Indications: Surgery-related factors	1	sNPWT is recommended for knee and hip arthroplasty surgeries exceeding 120 min.	A	88.9
	2	Revision surgeries involving implant removal are a standard indication for sNPWT.	B	100
Indications: Patient-related factors	3	sNPWT is recommended for patients with morbid obesity (BMI >40 kg/m^2^).	A	88.9
	4	sNPWT is recommended for patients with uncontrolled diabetes (HbA1c >8%).	A	88.9
	5	sNPWT can be routinely preferred in post-bariatric surgery patients undergoing arthroplasty.	C	77.8
Indications: Wound-related factors	6	sNPWT is recommended for use in high-tension wounds with less-than-appropriate primary closure.	B	100
Contraindications	7	sNPWT is contraindicated in wounds with high exudate output requiring dressing changes every 24 h or less.	B	88.9
	8	sNPWT is contraindicated in cases of known allergy to adhesives.	C	77.8
Application guidelines	9	sNPWT dressings should be applied with the knee at 90° flexion.	C	88.9
	10	The soft port of the sNPWT device should be positioned upwards for optimal drainage.	C	100
	11	Silicone-based adhesives are preferred over acrylic adhesives.	C	100
	12	Routine second sNPWT dressing changes are not necessary unless clinically indicated.	B	100

*Evidence Levels: Level A:* High-quality evidence supported by systematic reviews, meta-analyses, randomised controlled trials, or international clinical guidelines.

*Level B:* Moderate-quality evidence supported by prospective or retrospective comparative studies or consistent observational evidence.

*Level C:* Limited evidence based primarily on expert consensus, indirect evidence, biological rationale, or clinical experience.

sNPWT, single-use negative pressure wound therapy; BMI, body mass index; HbA1c, glycated haemoglobin.

*Clinical definitions*: High-tension wounds refer to incisions requiring closure under substantial soft-tissue tension or extensive mobilisation. Compromised primary closure refers to wound closure achieved with excessive tension, residual gapping, or concerns regarding tissue perfusion. Excessive wound exudate is defined as drainage requiring dressing replacement within 24 h despite appropriate wound closure. Persistent serous drainage refers to non-purulent wound drainage continuing beyond 72 h postoperatively. These definitions were developed by expert consensus to improve consistency in interpreting the consensus statements.

## 3 Results

### 3.1 Expert panel participation and consensus achievement

The consensus-building process included 10 experts in the exploratory meeting and nine experts in the subsequent consensus meeting. All nine participating experts completed the anonymous voting for each of the 13 candidate statements; therefore, all reported agreement percentages are based on a denominator of nine experts, with no missing voting data. Descriptive statistical analysis of the responses showed that 12 of the 13 candidate statements achieved the predefined consensus threshold of ≥75% agreement and were accepted as final consensus recommendations, whereas one statement failed to reach the predefined threshold and was rejected. The complete list of all 13 candidate statements, including the rejected statement, together with the detailed voting distribution (Strongly agree, Agree, Disagree, and Strongly disagree), overall agreement percentage, and consensus outcome, is provided in Table 4. The accepted consensus statements are summarised in Table 3.

**Table 4:** Summary of clinical evidence supporting consensus statements on sNPWT use in hip and knee arthroplasty.

Consensus statement/Risk factor	Study type & Reference	Key findings related to complication risk
Prolonged surgery (>120 min)	Meta-analysis (Scigliano NM et al., 2022) [14]	Procedures >120 min had significantly higher risk of PJI (OR: 1.63; 95% CI: 1.00–2.66; *p* = 0.048).
	Retrospective Analysis (Duchman KR et al., 2017) [15]	Surgery-related complications increased with operative time: 5.9% for >120 min vs. 4.6% for <60 min (*p* < 0.001).
Morbid obesity (BMI >40)	Retrospective Study (D’Apuzzo MR et al., 2015) [17]	Morbidly obese patients had higher risk of in-hospital infection post-TKA vs. non-obese (0.24% vs. 0.17%; OR: 1.3; *p* = 0.001).
	Systematic Review (McElroy MJ et al., 2013) [16]	Higher rate of post-TKA complications in obese (22%) and morbidly obese (15%) vs. non-obese (9%) patients.
Uncontrolled diabetes (HbA1c >8%)	Meta-analysis (Kong L et al., 2017) [18]	Diabetes increased risk of infection after TJA (OR: 1.58; 95% CI: 1.37–1.81).
	Retrospective Study (Wu C et al., 2014) [19]	Patients with diabetes had increased risk of post-arthroplasty infection (OR: 5.47; 95% CI: 1.77–16.97; *p* = 0.003).
Chronic kidney disease/Dialysis	Meta-analysis (Kim CW et al., 2020) [20]	Dialysis patients had higher risk of PJI (OR: 3.50; 95% CI: 1.54–7.95; *p* = 0.003) and revision (OR: 2.15; 95% CI: 1.77–2.62; *p* < 0.00001).
	Retrospective Study (Ponnusamy KE et al., 2015) [21]	Dialysis-dependent patients had higher rates of wound hematoma/seroma (THA: 3.78% vs. 1.19%) and infection (THA: 1.16% vs. 0.27%).
Coagulation disorders	Retrospective Study (Wang Y et al., 2022) [22]	Chronic thrombocytopenia increased complication risk post-THA (OR: 2.72) and post-TKA (OR: 2.55).
	Retrospective Study (Chung JJ et al., 2021) [23]	Bleeding disorders associated with higher risk of deep infection (OR: 2.49) and wound complications (OR: 1.59).
Hypoalbuminemia (<3.0–3.5 g/dL)	Retrospective Cohort (Bohl DD et al., 2016) [24]	Hypoalbuminemia associated with higher SSI risk (2.29% vs. 0.96%; adjusted RR: 2.0; *p* < 0.001) and overall complications.
	Retrospective Study (Walls JD et al., 2015) [25]	Hypoalbuminemia predicted higher risk of superficial SSI (OR: 2.61) and deep SSI (OR: 2.35) after THA.
High-tension wounds	Retrospective Clinical Study (Hsiao HY et al., 2022) [26]	NPWT group showed significant reduction in off-bed time (4.6 vs. 5.5 days; *p* = 0.028), indicating improved healing.
Hematoma/Seroma	Systematic Review (Norman G et al., 2022) [27]	NPWT showed a trend towards reducing seroma incidence (RR: 0.82; 95% CI: 0.65–1.05) compared to standard dressings.
	Prospective Randomized Study (Pachowsky M et al., 2012) [28]	Lower incidence of seroma in NPWT vs. standard dressing group (44% vs. 90%) after THA.

### 3.2 Consensus on indications for sNPWT

The expert panel reached a strong consensus on specific indications for the preemptive use of sNPWT, which were categorized into surgery-related, patient-related, and wound-related factors. Regarding surgery-related factors, two key indications were strongly endorsed. The expert panel agreed that prophylactic sNPWT may be considered for arthroplasty procedures exceeding 120 min (88.9% agreement) was established as a consensus-supported indication. The expert panel agreed that revision arthroplasty involving implant removal may be considered an appropriate indication for prophylactic sNPWT. For patient-related risk factors, the panel identified several high-risk comorbidities. The panel agreed that prophylactic sNPWT may be considered in patients with morbid obesity (BMI >40 kg/m^2^) and may be appropriate in patients with uncontrolled diabetes (HbA1c >8%), both achieving 88.9% agreement for sNPWT use. Furthermore, a consensus (77.7% agreement) was reached to routinely prefer sNPWT in patients undergoing arthroplasty following bariatric surgery. The panel also provided expert opinions on other significant risk factors, such as renal dialysis, immunosuppressant use, coagulation disorders, hypoalbuminemia (serum albumin <3.0 g/dL), and problematic local skin conditions. Concerning wound-related factors, unanimous consensus (100% agreement) was achieved that prophylactic sNPWT may be considered for high-tension wounds, defined as surgical incisions requiring closure under substantial soft-tissue tension or extensive tissue mobilisation, particularly where the operating surgeon considers the primary closure to be compromised because of excessive tension or reduced soft-tissue integrity. Examples discussed by the panel included complex revision arthroplasty, obese patients with thick, soft tissues, and wounds demonstrating significant tension during knee flexion. Additionally, the panel opined that wounds demonstrating persistent serous drainage beyond 72 h, recurrent dressing saturation, or postoperative haematoma requiring repeated dressing changes after exclusion of active infection were considered by the panel to represent situations in which prophylactic sNPWT may be appropriate.

### 3.3 Consensus on contraindications for sNPWT

The expert panel established clear contraindications for the application of sNPWT. A strong consensus (88.9% agreement) was reached that the panel agreed that prophylactic sNPWT should generally be avoided in wounds with high exudate output, defined as wound drainage that completely saturates the dressing or necessitates dressing replacement at intervals of 24 h or less despite appropriate wound closure. In such situations, the panel agreed that conventional canister-based negative pressure wound therapy may be more appropriate than single-use sNPWT.

Furthermore, a known allergy to adhesives was identified as a contraindication (77.8% agreement), noted as a strict precaution to prevent adverse skin reactions. Beyond these formal consensus statements, further expert discussion highlighted additional scenarios where sNPWT should be avoided, including the presence of eschar or necrotic tissue, untreated osteomyelitis, and situations where major vessels, nerves, or organs are exposed at the incision site.

### 3.4 Consensus on application guidelines for sNPWT

The panel established definitive best-practice guidelines for the application of sNPWT dressings to optimize efficacy and minimize complications. A strong consensus was achieved on four key principles. To ensure dressing adherence and prevent skin complications such as peeling and blisters, it was recommended (88.9% agreement) that the dressing be applied with the knee flexed at 90°. Unanimous consensus (100% agreement) was reached on positioning the device’s soft port in an upward, anti-gravity orientation to facilitate optimal drainage. Furthermore, the panel unanimously favoured silicone-based adhesives over acrylic alternatives (100% agreement) due to their superior skin adhesion and reduced risk of irritation. Finally, it was unanimously agreed (100% agreement) that a routine second dressing change is not necessary and should only be performed if clinically indicated, thereby minimizing unnecessary disruption to the wound environment.

Expert opinion further recommended using non-residual, alcohol-based solutions or saline for skin preparation, avoiding betadine, to ensure optimal adhesive contact. A clinical decision pathway synthesizing these consensus statements and expert opinions is presented in Figure 2.

## 4 Discussion

This expert consensus study proposes consensus-based recommendations of India-specific recommendations for the preemptive use of sNPWT on closed incisions following total hip and knee arthroplasty. The consensus achieved across 12 key statements underscores a unified approach to standardizing practice and mitigating the risk of surgical site complications in a diverse patient population. The recommendations strategically address three critical pillars: patient selection (indications), safety (contraindications), and technical execution (application guidelines).

The strong consensus on using sNPWT for revision surgeries and procedures exceeding 120 min reflects the heightened risk of SSIs in complex cases, a view supported by international expert panels that identify prolonged operative time as a key risk factor and periprosthetic joint infection [PJI] [9–11]. By formally endorsing sNPWT for these scenarios, the panel provides a clear, evidence-based protocol for high-risk procedures. More significantly, the consensus on patient-related risk factors including morbid obesity [BMI >40 kg/m^2^], uncontrolled diabetes [HbA1c >8%], and post-bariatric surgery status directly addresses the unique comorbidity profile often encountered in Indian arthroplasty patients. This aligns with the study’s core objective of tailoring international guidelines to local epidemiological realities, ensuring that prophylactic interventions are targeted effectively within resource-conscious frameworks [12, 13].

The unanimous agreement on using sNPWT for high-tension wounds acknowledges the biomechanical benefits of the therapy. This consensus is crucial, as it provides an objective criterion for application beyond systemic patient factors, focusing on the local wound environment’s integrity. The contraindications established by the panel, particularly for high-exudate wounds and adhesive allergies, are equally important. These guidelines prevent the inappropriate use of sNPWT in situations where it is likely to be ineffective or harmful, thereby promoting patient safety and optimizing resource utilization.

Another important aspect highlighted during the consensus discussions was the frequent misconception surrounding the terminology and indications of negative pressure wound therapy in surgical practice. Traditional vacuum-assisted closure therapy has historically been used for the management of open wounds, infected wounds, and complex soft-tissue defects. In contrast, sNPWT, as discussed in this consensus, is intended for prophylactic application on closed surgical incisions to reduce postoperative wound complications. These approaches differ in their indications, device design, and therapeutic objectives. Confusion between these modalities may lead to inappropriate expectations or underutilization of prophylactic sNPWT in high-risk surgical incisions. Clear differentiation between therapeutic SNPWT for open wounds and prophylactic incisional SNPWT for closed surgical wounds is therefore important for appropriate clinical adoption.

The detailed application guidelines formulated by the panel are a key contribution of this consensus. Recommendations such as applying the dressing at 90° of knee flexion, positioning the soft port upwards, and preferring silicone-based adhesives are rooted in practical clinical experience. These nuances are rarely detailed in international guidelines but are critical for ensuring the therapy’s success in daily practice. The unanimous agreement against routine second dressing changes reinforces a “do not disturb” philosophy, minimizing unnecessary wound manipulation and potential contamination.

An important contribution of the present consensus is the introduction of practical operational definitions for commonly encountered clinical scenarios that have previously been described inconsistently in the literature. Terms such as high-tension wound, compromised primary closure, persistent serous drainage, and excessive wound exudate are frequently used in clinical practice but lack universally accepted definitions. By providing clinically applicable descriptions and representative examples, the panel sought to improve consistency in patient selection and facilitate more uniform application of prophylactic sNPWT across different clinical settings. Nevertheless, these definitions should be interpreted as pragmatic guidance rather than absolute thresholds, and individual clinical judgement remains essential when managing complex patients.

When viewed alongside recommendations from bodies like the WHO and NICE [3, 4], this consensus complements global standards by adding granular, context-specific layers. While international guidelines support consideration of prophylactic sNPWT for selected high-risk patients and surgical incisions, they do not recommend its routine application following all hip and knee arthroplasty procedures. The present consensus complements these recommendations by providing practical guidance on patient selection and application within the Indian clinical setting. The developed clinical decision pathway (Figure 2) serves as a practical tool for surgeons to integrate these consensus-informed recommendations into their clinical workflow (Table 2). Although these recommendations were developed for the Indian clinical setting, several identified indications and technical considerations are consistent with existing international evidence. The present work should therefore be viewed as a complementary, context-specific framework that may inform future multinational consensus initiatives aimed at harmonising recommendations across different healthcare systems.

An additional consideration in the Indian healthcare setting is the economic impact of prophylactic sNPWT. Although international studies have suggested that selective use of sNPWT in high-risk patients may reduce postoperative wound complications, hospital readmissions, and revision procedures, its upfront device cost may limit widespread adoption, particularly in resource-constrained healthcare systems. Furthermore, reimbursement policies for prophylactic sNPWT vary across public and private healthcare providers, and routine reimbursement is not universally available in India. Consequently, the expert panel agreed that these consensus recommendations should support a risk-based patient selection strategy rather than routine application in all arthroplasty procedures. Appropriate selection of patients with recognised risk factors may improve resource utilisation while minimising unnecessary costs and potential overuse in low-risk individuals. Future economic evaluations performed within the Indian healthcare system are required to determine the cost-effectiveness, budget impact, and value of prophylactic sNPWT across different patient populations and practice settings.

**Figure 3:**
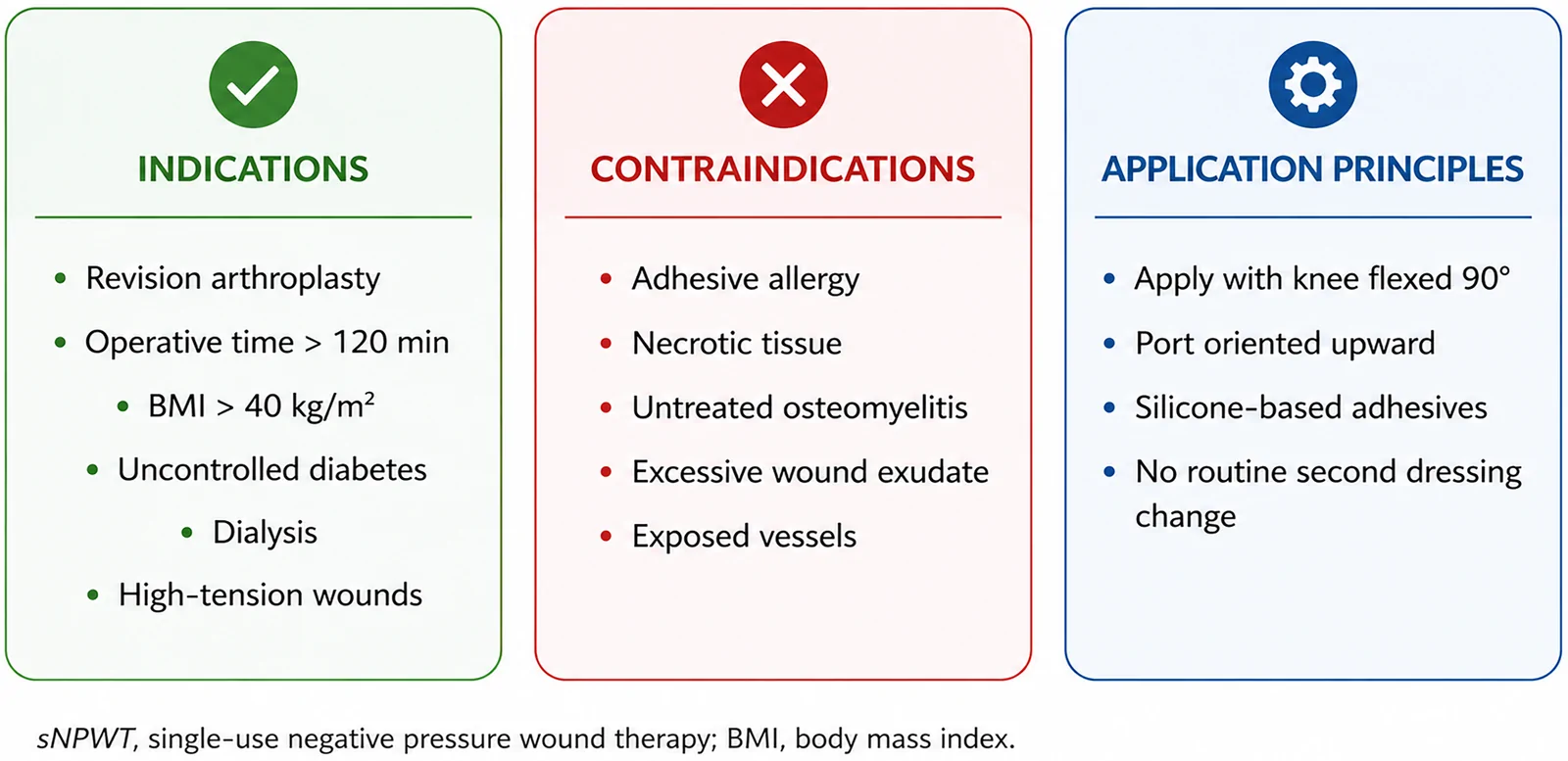
Summary of consensus recommendations for sNPWT.

The recommendations presented in this study should be interpreted within the context of a modified Delphi consensus methodology. Although the consensus statements were informed by the best available published evidence and extensive clinical experience, they do not constitute definitive evidence of clinical effectiveness. Rather, they represent expert-informed clinical guidance intended to support decision-making in areas where arthroplasty-specific evidence remains limited. The panel agreed that prophylactic sNPWT may be considered in carefully selected clinical scenarios based on individual patient characteristics, surgical factors, surgeon judgement, institutional protocols, and the evolving evidence base. These recommendations are intended to complement, rather than replace, existing clinical guidelines and evidence-based practice (Figure 3).

The present consensus should therefore be viewed as a framework to guide clinical decision-making rather than evidence that implementation of these recommendations will improve patient outcomes or reduce postoperative complications. Such benefits remain to be confirmed through appropriately designed prospective clinical studies.

### 4.1 Limitations

This study has several limitations. The recommendations are based on expert consensus rather than prospective comparative clinical studies or randomized controlled trials. Although consensus methodology is valuable where evidence is limited or heterogeneous, expert opinion represents a lower level of evidence than high-quality clinical research. The panel consisted exclusively of experienced arthroplasty surgeons practicing in India. While this was consistent with the objective of developing context-specific recommendations, the findings may not be generalisable to healthcare systems with different patient populations, surgical practices, and resource availability. The formal evidence grading using established frameworks such as GRADE was not performed. Instead, consensus statements were developed through expert interpretation of the available literature and clinical experience. The clinical effectiveness, cost-effectiveness, and budget impact of prophylactic sNPWT were not evaluated in this study. No formal health economic analysis was performed, and therefore the affordability, reimbursement, device availability, and resource implications of implementing prophylactic sNPWT across different healthcare settings in India could not be assessed. Because the recommendations were developed to support selective use in high-risk patients rather than routine application in all arthroplasty procedures, future prospective studies should incorporate cost-effectiveness analyses, reimbursement modelling, budget impact assessments, and resource utilisation outcomes to better inform implementation within diverse healthcare systems.

The present recommendations should be interpreted as consensus-informed clinical guidance derived from expert opinion integrated with the best available published evidence. They are intended to support clinical decision-making in areas where arthroplasty-specific evidence remains limited and should not be interpreted as formal evidence-based clinical practice guidelines.

## 5 Conclusion

This modified Delphi consensus provides evidence-informed, expert recommendations for the selective use of prophylactic sNPWT in hip and knee arthroplasty within the Indian clinical setting. By defining patient selection criteria, contraindications, and practical application principles, these recommendations aim to support consistent, evidence-informed clinical decision-making and facilitate the appropriate implementation of sNPWT in routine arthroplasty practice. While further prospective multicentre studies, randomized controlled trials, and health economic evaluations are warranted to validate and refine these recommendations, the present consensus offers a pragmatic framework for translating current evidence and collective clinical experience into everyday practice.

## Abbreviations

sNPWT: single-use negative pressure wound therapy
NPWT: negative pressure wound therapy
SSI: surgical site infection
PJI: periprosthetic joint infection
THA: total hip arthroplasty
TKA: total knee arthroplasty

## Data Availability

Data availability is not applicable.
